# Using Entropy for Welds Segmentation and Evaluation

**DOI:** 10.3390/e21121168

**Published:** 2019-11-28

**Authors:** Oto Haffner, Erik Kučera, Peter Drahoš, Ján Cigánek

**Affiliations:** Faculty of Electrical Engineering and Information Technology, Slovak University of Technology in Bratislava, 841 04 Bratislava, Slovakia; erik.kucera@stuba.sk (E.K.);

**Keywords:** weld segmentation, local entropy filter, weld evaluation, convolution neural network, image entropy, Python, Keras, RSNNS, MXNet

## Abstract

In this paper, a methodology based on weld segmentation using entropy and evaluation by conventional and convolution neural networks to evaluate quality of welds is developed. Compared to conventional neural networks, there is no use of image preprocessing (weld segmentation based on entropy) or data representation for the convolution neural networks in our experiments. The experiments are performed on 6422 weld image samples and the performance results of both types of neural network are compared to the conventional methods. In all experiments, neural networks implemented and trained using the proposed approach delivered excellent results with a success rate of nearly 100%. The best results were achieved using convolution neural networks which provided excellent results and with almost no pre-processing of image data required.

## 1. Introduction

The Fourth Industrial Revolution (Industry 4.0) has opened space for research and development of new manufacturing methods, systems and equipment based on innovations such as computing intelligence, autonomous robots, big data, augmented reality, process simulation, quality management systems, etc. [1].

Weld evaluation is very important quality control process in many manufacturing processes. Without this technological process, it would be almost impossible to produce welded constructions with current efficiency—whether we are talking about time, price, or material consumption. It is therefore necessary to welds be inspected to meet the specified quality level. In order to detect the possible presence of different weld defects, proper sensing, monitoring and inspection methods are necessary for quality control. Very effective and non-destructive method for weld evaluation is visual inspection. Inspection process using this method can be in certain level automated and done by computer systems [2,3].

Visual inspection of a weld is an important non-destructive method for weld quality diagnostics that enables to check welded joint and its various parameters. This examination is carried out as a first examination and able to detect various defects [4].

In this paper, we focus on indirect visual evaluation due to which the evaluation process can be automated. Indirect inspection can be applied also in places that are not directly accessible, for example the inner surface of a pipeline, the interior of pressure vessels, car body cavities etc. It also eliminates errors of human judgment and removes errors caused by workers for such reasons as e.g., fatigue, inattention or lack of experience.

The improved beamlet transformation for weld toe detection described in [5,6] considers images which are corrupted by noise. The authors aim at detecting edge borders of welds. The dynamic thresholding is performed in one of the beamlet algorithm steps. The algorithm predicts the directional characteristics of the weld allows to filtrate unsuitable edges. Using this method, it is possible to directly extract weld seam edges from highly noisy welding images without any pre-processing or post-processing steps.

In [7], the authors work with pipeline weld images with a very low contrast and corrupted by noise; this causes problems to conventional edge detectors. At first, the image is noise-filtered using a morphological operation of opening and closing. Next, the improved algorithm of fuzzy edge detection is applied. Multi-level fuzzy image improvement is based on interactive searching of optimal threshold level and multi-directional edge detector which convolution kernel is 5 × 5 with 8 directions based on gradient searching. The result of the algorithm is compared with detectors as Sobel, canny FED and fast FED.

Edge detection and histogram projection are used in [8], where histogram projections of tested welds are compared with a specified similarity threshold used to evaluate quality of the tested welds. The loaded image pattern has the same specifications (width and position) as the tested image. Always one vertical line from the pattern and the tested images is compared. Line histograms of pattern and tested images are computed, the correlation degree of two histograms is computed using the Tukey HSD difference. A lower correlation degree than the specified correlation threshold indicates edge defects in this part of the examined image. The procedure is repeated over the entire width of image.

Evaluation of metal cans welds is dealt with in [9]. Can’s weld defects may not be directly related to welding (they can be brought about by rest of glue, dust, etc.). Therefore, authors use probability evaluation of two evaluation methods; the Column Gray-Level Accumulation Inspection represents histogram projection in general. The histogram projections of the pattern and the tested weld are compared. The comparison of first derivation for making better results is also performed. This method can detect defects of wider surface. The overall evaluation is done using Dampster-Shafer theory of evidence.

In another work [10], the above authors deal with edge detection based on pixel intensity difference of the foreground and the background. The background pixels’ intensity occurs with a maximum probability and the distribution of the background pixels fits the Gauss distribution.

The weld visual inspection process performed through image processing on the image sequence to improve data accuracy is presented in [11]. The Convolution Neural Network (CNN) as an image processing technique can determine the feature automatically to classify the variation of each weld defect pattern. A classification using CNN consists of two stages: image extraction using image convolution, and image classification using neural network. The proposed evaluation system has obtained classification for four different types of weld defects with validation accuracy of 95.83%.

A technique for automatic endpoint detection of weld seam removal in a robotic abrasive belt grinding process using a vision system based on deep learning is demonstrated in [12]. The paper presents results of the first investigative stage of semantic segmentation of weld seam removal states using encoder-decoder convolutional neural networks (EDCNN). The prediction system based on semantic segmentation is able to monitor weld profile geometry evolution taking into account the varying belt grinding parameters during machining which allows further process optimization.

Utilizing computing intelligent using support vector machine (SVM) is presented in [13,14]. Authors developed real-time monitoring system to automatically evaluate the welding quality during high-power disk laser welding. Fifteen features were extracted from images of laser-induced metal vapor during welding. To detect the optimal feature subset for SVM, a feature selection method based on the SFFS algorithm was applied. An accuracy of 98.11% by 10-fold cross validation was achieved for the SVM classifier generated by the ten selected features. The authors declare the method has the potential to be applied in the real-time monitoring of high-power laser welding.

The authors of [15,16,17,18] deal with the development of a system for automatic weld evaluation using new information technologies based on cloud computing and single-board computer in the context of Industry 4.0. The proposed approach is based on using a visual system for weld recognition, and a neural network cloud computing for real-time weld evaluation, both implemented on a single-board low-cost computer. The proposed evaluation system was successfully verified on welding samples corresponding to a real welding process. The system considerably contributes to the weld diagnostics in industrial processes of small- and medium-sized enterprises. In [18], the same authors use a single-board computer able to communicate with an Android smartphone which is a very good interface for a worker or his shift manager. The basic result of this paper is a proposal of a weld quality evaluation system that consists of a single-board computer in combination with Android smartphone.

This paper deals with development of a software system for visual weld quality evaluation based on weld segmentation using entropy and evaluation by conventional and convolution neural networks. The evaluation of the performance results is compared to the conventional methods (weld segmentation based on entropy and evaluation using conventional neural networks with and without weld segmentation). Most experiments of proposed method apply on weld metal, however, one experiment with convolution neural networks applies also on weld adjected zones. 6422 real and adjusted laboratory samples of welds are used for experiments. The paper is organized in five sections: Section 2 deals with preparation of input data for the neural network. Section 3 describes configuration of used neural networks and their training process. In Section 4 the results of experiments are presented. In Section 5 we discuss the results.

## 2. Preparation of Input Data for the Neural Network

**Figure 1:**
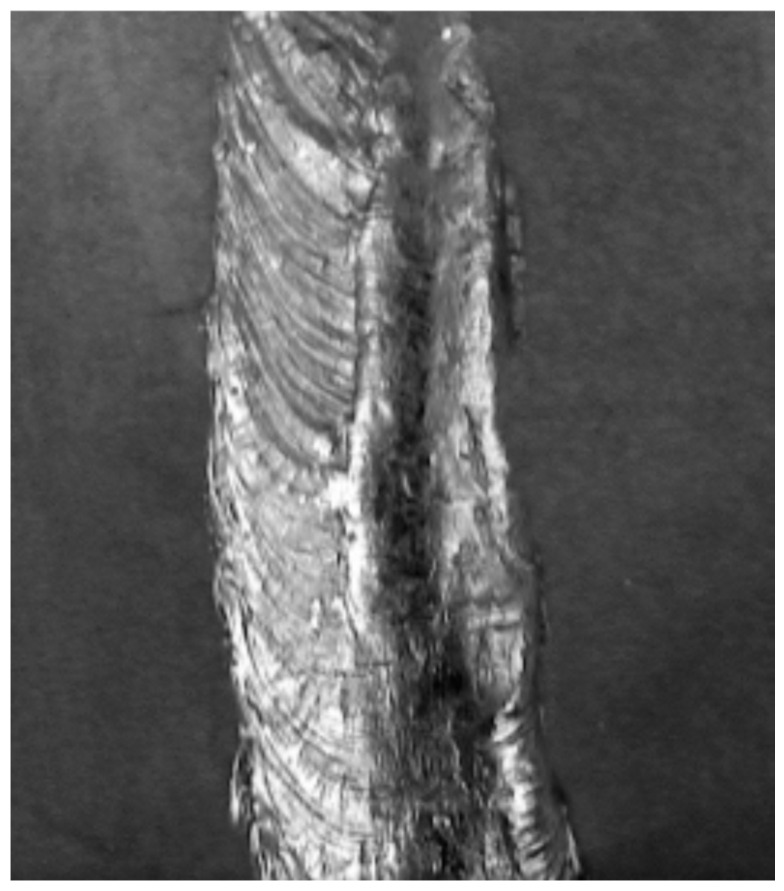
Laboratory sample of an OK weld.

**Figure 2:**
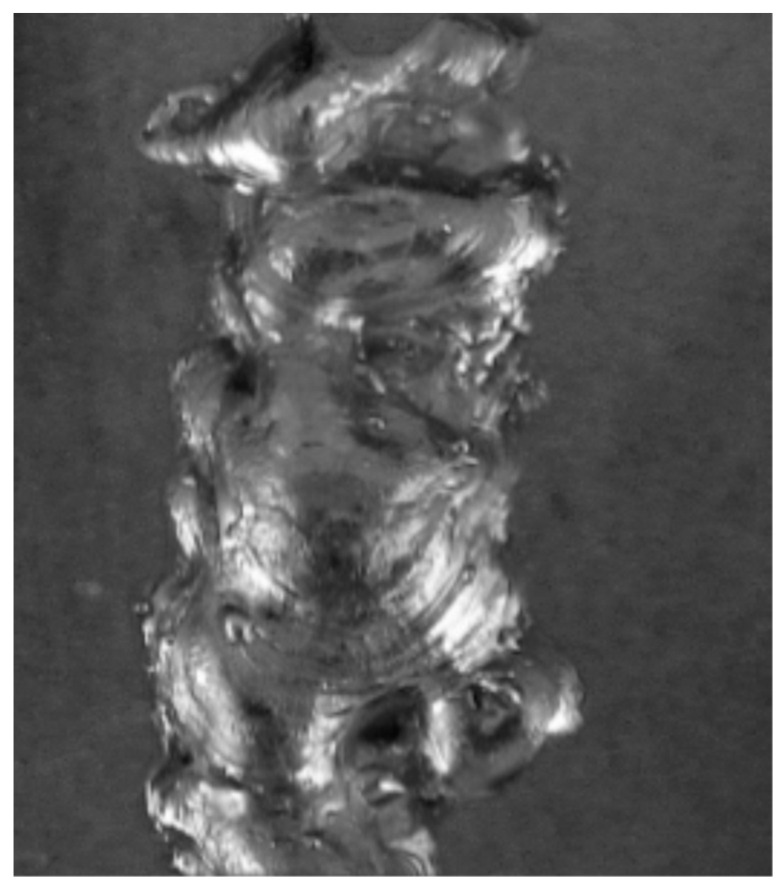
Laboratory sample of NOK weld.

The input data for the proposed diagnostic system were represented in the form of grayscale laboratory samples of metal sheet welds in JPEG format. The samples were pre-classified as OK (correct) and NOK (incorrect) (Figure 1 and Figure 2). Defective weld samples (NOK) include samples of various surface defects such as irregular weld bead, excess weld metal, craters, undercut, etc. Welds images are captured under the same illumination and have the same resolution 263 × 300 pixels. The total number of evaluated sample images was 6422.

However, for several reasons the image resolution 263 × 300 pixels is not suitable for a conventional neural network due to the necessity of large amount of allocated memory (about gigabytes for thousands of frames even in a relatively low resolution) and time-consuming network training time.

Several suitable options for data processing that eliminate the above problems are presented next. At first, the background weld segmentation is described. Segmentation provides two outputs - the weld mask and the segmented weld itself. Three transformations of the weld mask into a one-dimensional feature vector are described further. Feature vectors are useful as inputs for the multilayer perceptron (MLP)/radial basis function (RBF) neural networks. Finally, the size of the segmented/unsegmented weld image is reduced when applied in the conventional neural network (if CNN is applied, no size reduction is needed).

### 2.1. Weld Segmentation

**Figure 3:**
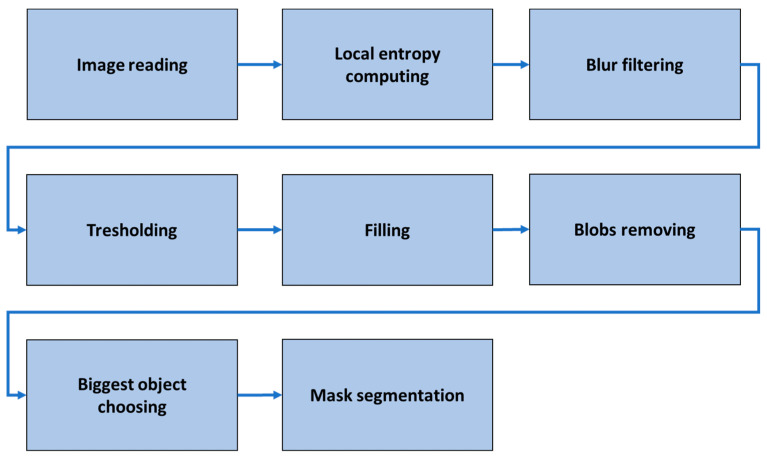
The simplified flowchart of the segmentation algorithm.

The sample images depict the weld itself and the background—metal sheet. The background does not affect the evaluation of the weld and is masked from the images by the proposed algorithm. The simplified flowchart of the algorithm is shown in Figure 3.

After reading the images, local entropy of each pixel is computed according to [19]:

(1) $$\overset{K}{\underset{i=1}{\sum }}\overset{K}{\underset{j=1}{\sum }}p_{ij}\log_{2}p_{ij},$$

where $p_{ij}$ represents the probability function for the pixel [i,j].

**Figure 4:**
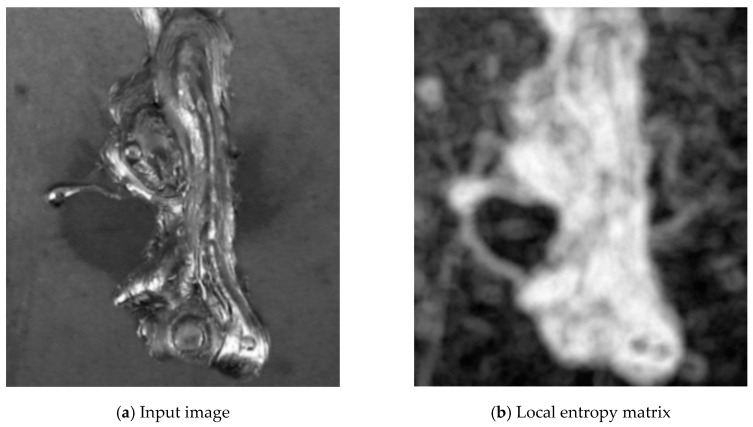
Step 1—local entropy computing.

This value contains information about the complexity/unevenness around the pixel. The neighbourhood radius was set to 8 pixels. To compute the entropy, the filters.rank.entropy function from the Python library scikit-image was used. The resulting local entropy matrix effectively finds the edges and texture complexity in the image. The results of filtering can be seen in Figure 4.

**Figure 5:**
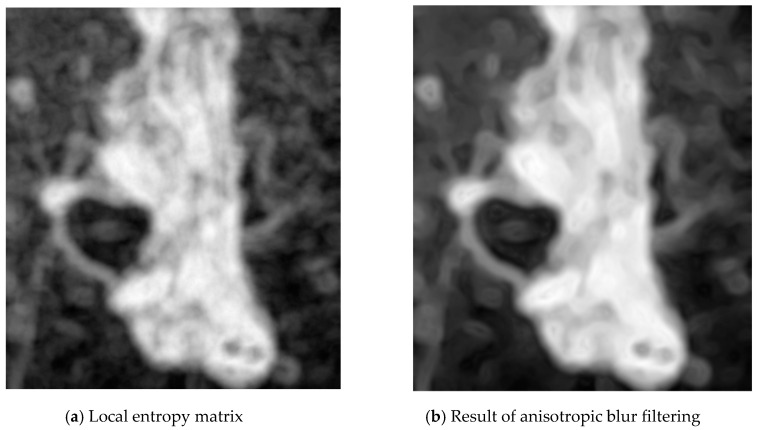
Step 2—blur filtering.

As the entropy resolution values were too detailed for our application, the blur filtering was applied. The anisotropic blur filter from the imager library was implemented, which removes noise/unimportant details while preserving edges better than other types of blur filters. The blur filter with an amplitude of 250 was applied (Figure 5).

**Figure 6:**
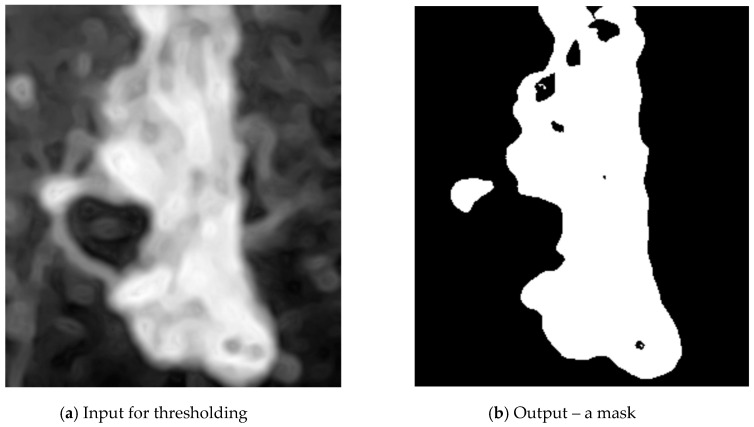
Step 3—thresholding.

The next step is thresholding. In the image matrix, the value 1 (white) represents weld pixels, the value 0 (black) represents background. Thresholding was implemented using the function threshold from the imager library. The optimal threshold value was computed automatically using the kmeans method (Figure 6).

**Figure 7:**
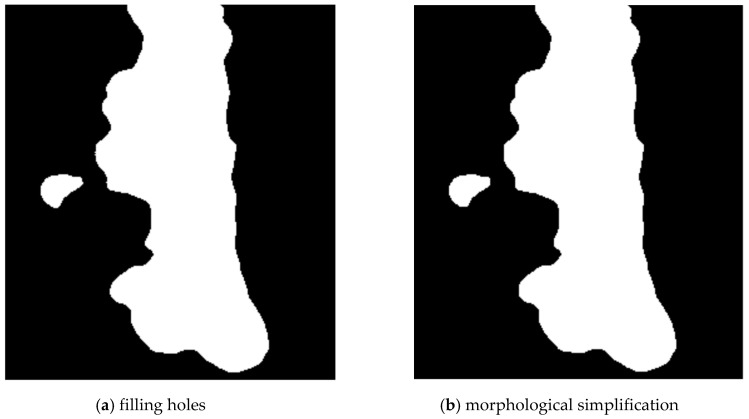
Step 4—filling holes (**a**) and morphological simplification (**b**).

The thresholding result may have some imperfections—small blobs and unfilled areas. Unfilled areas are removed using the inverted output of the function bucketfill (imager library). It is applied on the background of the weld and it finds all pixels of the background. The remaining the pixels are filled with value 1 (white) (Figure 7a).

Very small blobs were removed using the function clean (imager library). This function reduces objects size using morphological erosion, and then increases it. This causes, that very small objects are removed and the shape of larger object is simplified (Figure 7b).

**Figure 8:**
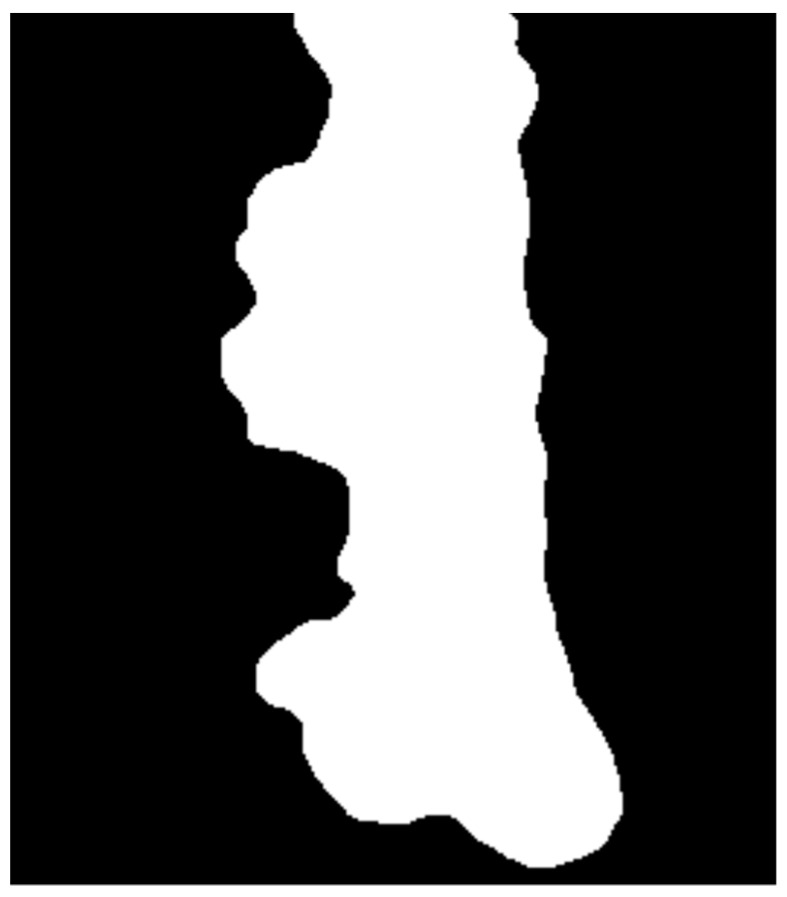
Step 5—Finding the largest object.

However, larger blobs were not removed in the previous step. To find the largest object in the image, the function split_connected (imager library) was used (Figure 8).

**Figure 9:**
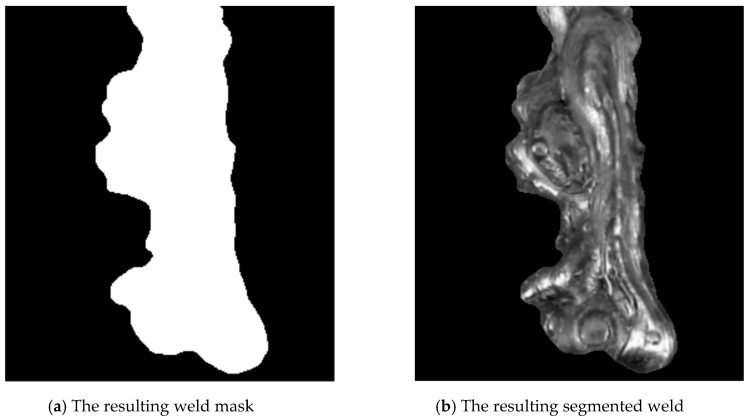
Results of segmentation.

The segmentation result—the mask and the masked weld can be seen in Figure 9.

### 2.2. Vector of Sums of Subfields in the Mask

The first representation of the mask is a vector which entries are sums of subfields. For input images of resolution 263 × 300 pixels, was selected a subfield of 50 × 50 pixels, which corresponds to 36 values. The function for vector calculation is shown in the Algorithm 1.

**Figure 10:**
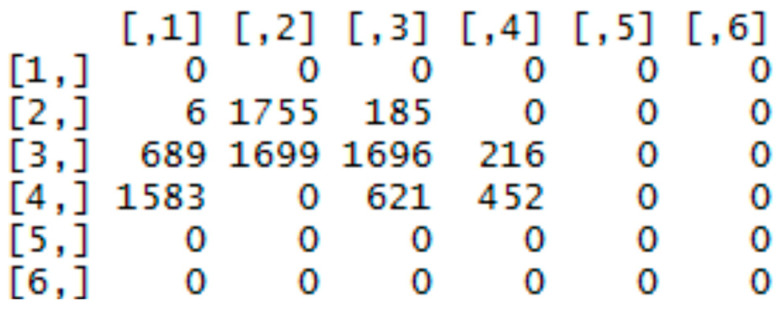
Two-dimensional array of sums.

**Figure 11:**
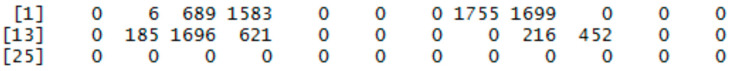
Resulting vector of sums.

The function ceiling rounds a number to the next higher integer. Using division of the index (i,j) by the size of the subfield, and subsequently the function ceiling, we obtained indI/indJ for the selected index i/j. The function as.vector retypes the resulting two-dimensional array into a vector by writing the matrix elements column-wise into a vector. Example of retyping can be understood from Figure 10 and Figure 11.

**Figure 12:**
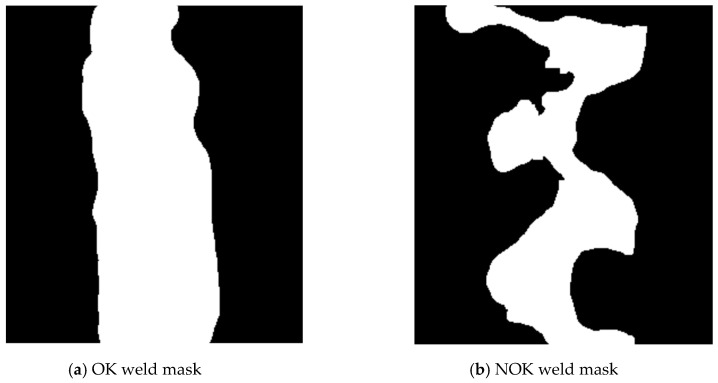
Weld masks.

**Figure 13:**
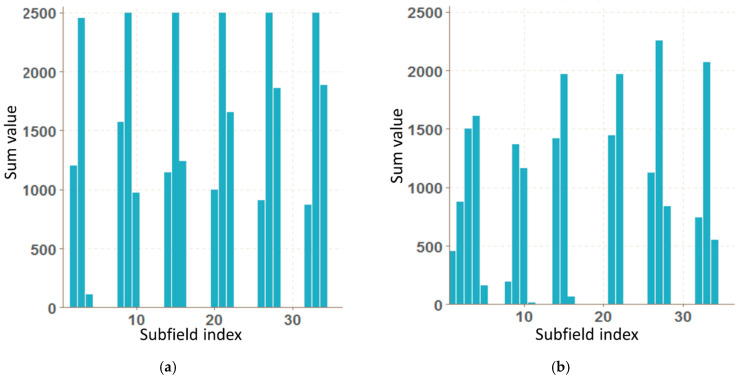
Graphs of vector of sums of subfields in the mask for OK (**a**) and NOK (**b**) weld.

Graphs for OK and NOK welds (Figure 12) can be compared in Figure 13: the OK mask graph has every third value (representing the subfields in the image center) maximal. Values of the NOK weld graph are distributed into more columns and the values do not achieve maximum values. The main drawback of this representation is that it can be used only for images with the same size. The benefit is a multiple reduction of input data (number of mask pixels in our case has been reduced 50^2^-times).

**Algorithm 1.** Computing of subfields sums of the mask**procedure** MaskToSums(img, size)  xLen ←length(img[ ,1])  yLen ←length(img[1, ])  nRows ← ceiling(xLen/size)  nCols ← ceiling(yLen/size)  res ← matrix(0, nRows, nCols)  **for** i **in** 1:xLen **do**    **for** j **in** 1:yLen **do**     **if** img[i,j] == TRUE **then**       indI ← ceiling(i/size)       indJ ← ceiling(j/size)       res[indI, indJ] ++     **end if**    **end for**  **end for**  **return** as.vector(res) **end procedure**

### 2.3. Histogram Projection of the Mask

**Figure 14:**
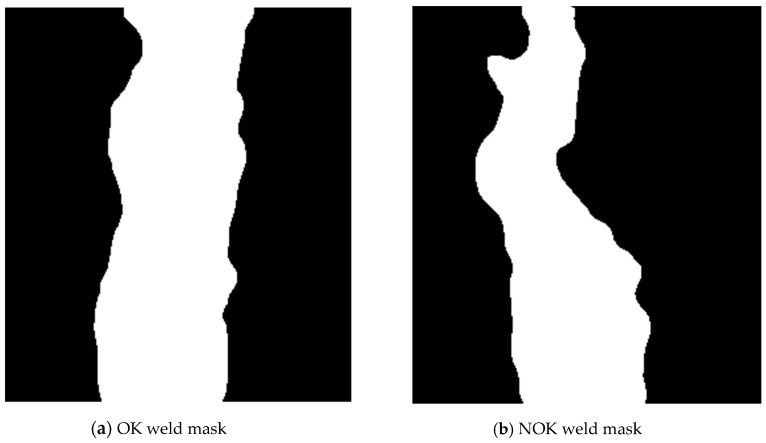
Weld masks for histogram projection.

A histogram projection is a vector containing sums of columns and rows of the input image matrix (Figure 14). In the case of an image mask, these are amounts representing numbers of white pixels. Thus, the length of the vector corresponds to the vector of the height and width of the image.

**Figure 15:**
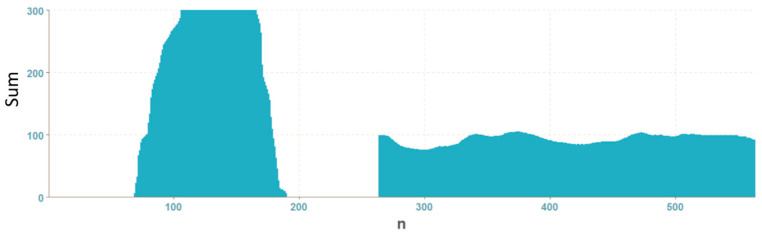
Graph of histogram projection of an OK weld.

**Figure 16:**
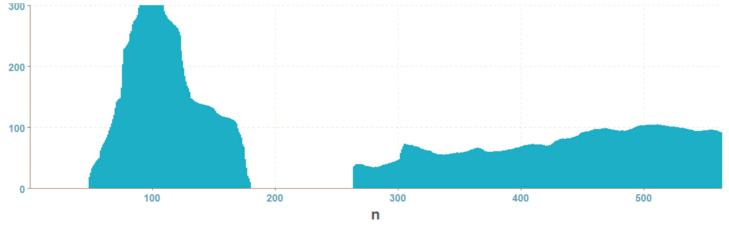
Graph of histogram projection of a NOK weld.

In the graphs (Figure 15 and Figure 16) showing the histogram projection of the mask, the difference between correct and wrong welds is visible. The projection of the correct weld mask is more even, the sums by columns have an even increase and slope, and the sums per line have small variations. On the other hand, the histogram projection of the wrong weld mask has a lot of irregularities. The disadvantage of this representation consists in that it cannot be used for input images of different resolutions. The resulting projection vector is much larger than other representations. The advantage is easy implementation and calculation.

### 2.4. Vector of Polar Coordinates of the Mask Boundary

A next representation of a weld mask in this paper is the vector of polar coordinates of the mask boundary. To transform weld masks, an algorithm has been proposed and implemented. Its main steps are described below.

The first step is to find the x, y coordinates of the mask boundary using the function boundary (imager library). Then, coordinates of the center of the object [cx, cy] are calculated according to:

(2) $$c_{x}=\frac{max\left(x\right)-min\left(x\right)}{2}+min\left(x\right),$$

(3) $$c_{y}=\frac{max\left(y\right)-min\left(y\right)}{2}+min\left(y\right),$$

In the next step, the position of the object is normalized (the center is moved to the position [0,0]) according to the found coordinates. Then, for each boundary point, the coordinates are converted from Cartesian to polar [r,α] (i.e., distance from center, angle). According to the Pythagorean theorem, the distance is calculated as follows:

(4) $$r=\sqrt{x^{2}+x^{2}},$$

Calculation of the angle is realized by Algorithm 2:

**Algorithm 2.** Calculation of angle from Cartesian coordinates**procedure** Angle(x, y)   z ← x + 1i * y   a ← 90 - arg(z) / π * 180   return round(a mod 360) **end procedure**

**Figure 17:**
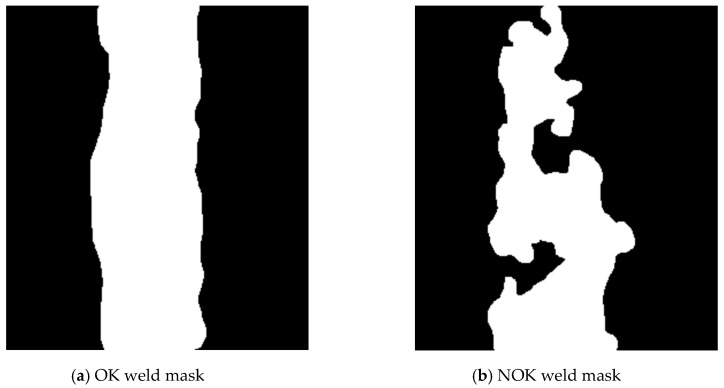
Mask of OK and NOK weld.

**Figure 18:**
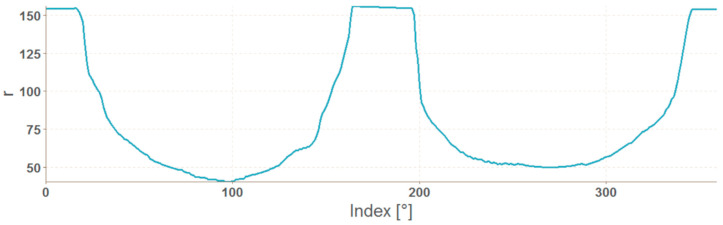
Graph of polar coordinates vector of an OK weld mask.

**Figure 19:**
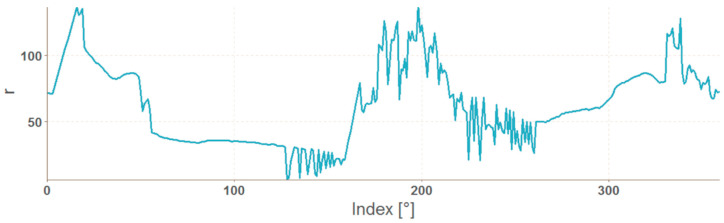
Graph of polar coordinates vector of a NOK weld mask.

If the resulting number of coordinates is less than 360, the missing angle values are completed and the corresponding distances are calculated from the surrounding values by linear interpolation using the na_approx function (zoo library). The result is a vector with 360 elements, which indices correspond to the angle values in degrees, and the value is the distance r. The resulting graphs of OK and NOK weld masks (Figure 17) are in Figure 18 and Figure 19.

The representation in the form of polar coordinates for the OK weld visibly differs from the NOK one. The big jumps and variations on the graph are caused by large irregularities in the weld shape. The advantage of such representation is that it can be used for any input mask resolution. The disadvantage is a complicated calculation. Generally, mask representations contain information only about the shape of the weld, which can be considered as a disadvantage because texture information is important input data for the neural network.

### 2.5. Data Preparation for Neural Network

Weld images and feature vectors were stored in two data structures of type list. The first list represented welds classified as NOK (incorrect); the second list welds classified as OK (correct). For neural networks, it was necessary to combine data, i.e., to transform and randomly mix them. For MLP and RBF networks, each input vector has to have assigned a classification value 0 (incorrect) or 1 (correct). Then, the vectors were merged together and with randomly mixed elements. Next, the L2-normalization was applied to the data. Finally, 85% of training and 15% of test samples were selected randomly. For convolution neural networks, the images were 5-times reduced, then the data type was converted to a three-dimensional array data structure. In the arrays, the dimensions were transposed to represent to correspond to the following structure: [number of images∗length∗height]. The vector of zeros with the same length as the first dimension corresponded to the first array (array of NOK welds). The vector of ones corresponded to the second array (array of OK welds). The arrays and vectors were merged into a common list and their elements were mixed randomly. Then, 77% of training samples, 15% of test samples and 8% of validation samples were selected.

## 3. Configuration and Training of Neural Networks

Several neural network architectures were configured for comparison and testing. Their parameters were changed during the experiments and the experiment results were compared and evaluated. Both RBF and MLP networks were configured in The Stuttgart Neural Network Simulator for R language - RSNNS library, the MLP networks were configured in the Keras library, and the convolution networks were configured in the Keras and the MXNet libraries.

### 3.1. RBF Network

**Figure 20:**
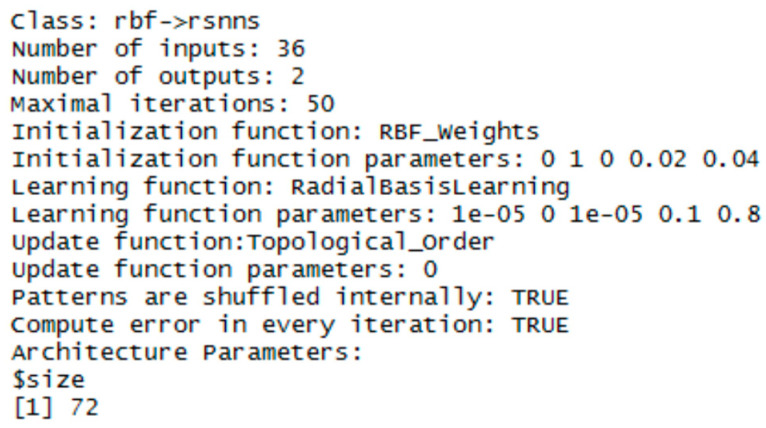
Settings for RBF network—for the vector of sums of subfields in the mask.

**Figure 21:**
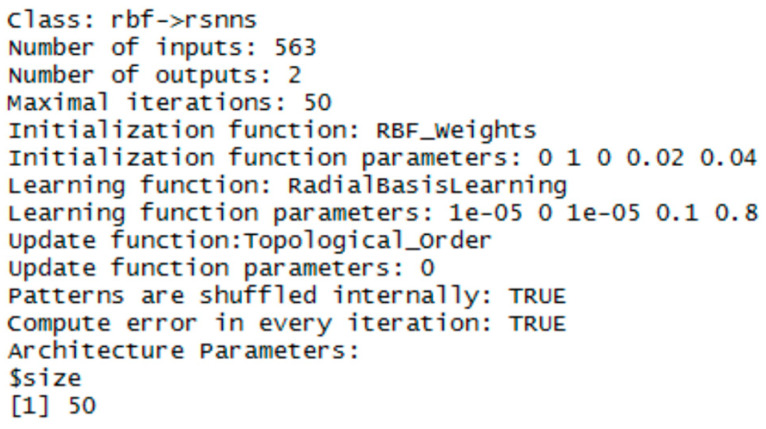
Settings for RBF network—for the histogram projection vector.

**Figure 22:**
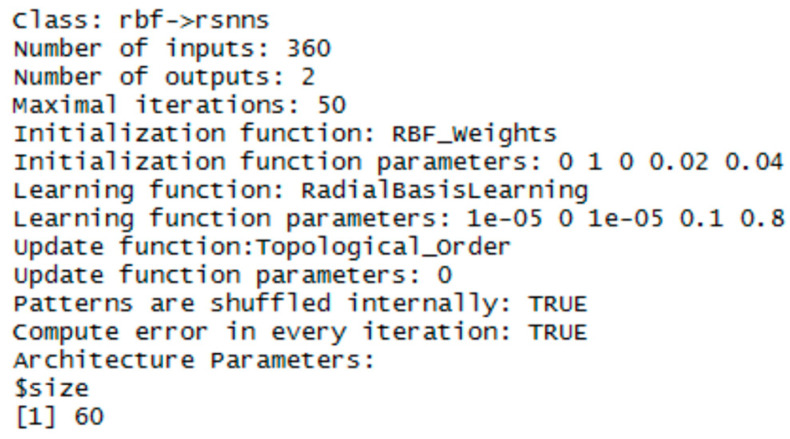
Settings for RBF network—for the polar coordinates vector.

To implement the RBF network, the RSNNS library was chosen (just in this one the RBF network template is available). Three RBF networks were configured using the function rbf (RSNN library). The set parameters were the number of units in the hidden layer and the number of epochs, the initial parameters had default values. The best configurations were chosen experimentally. Configuration details are in Figure 20, Figure 21 and Figure 22.

### 3.2. MLP Network

Experiments with training and testing of MLP networks showed, that a one-layer architecture is sufficient for our data representation. The performance of the network was very good and the difference from multiple hidden layers was negligible. To keep the objectivity, MLP networks had the same configuration in both libraries. The sigmoid activation function and the randomize weights initialization functions were used. For the NN training, the error backpropagation algorithm with learning parameter 0,1 was used.

**Figure 23:**
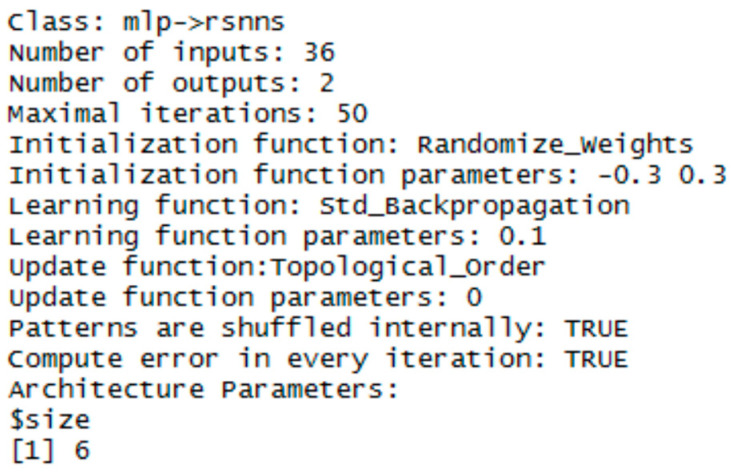
Settings for MLP network—for vector of sums of subfields in the mask.

**Figure 24:**
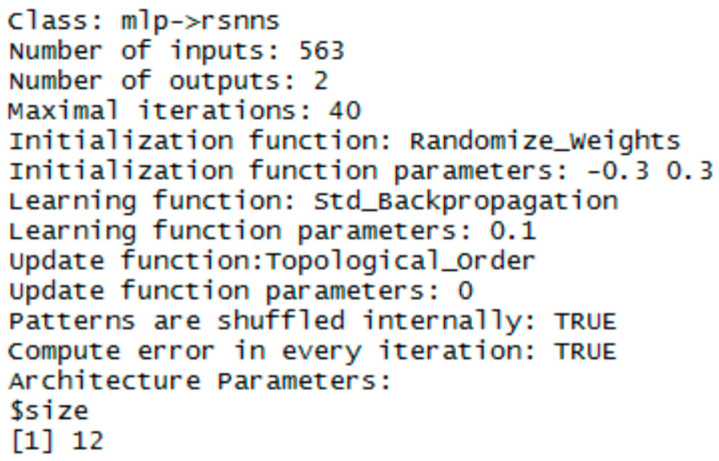
Settings for MLP network—for histogram projection vector.

**Figure 25:**
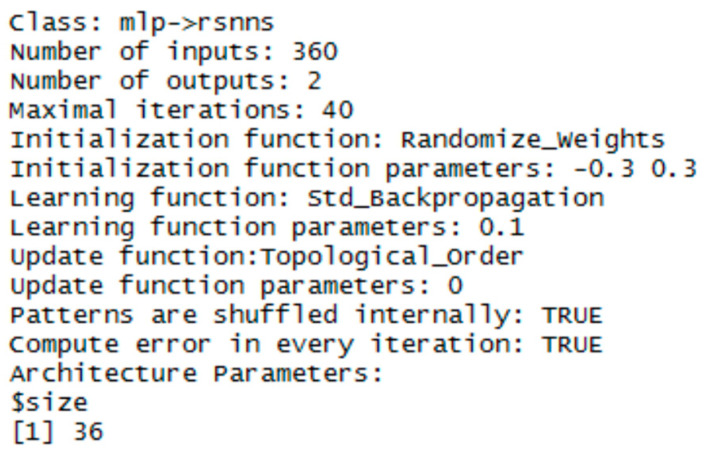
Settings for MLP network - for the polar coordinates vector.

The implementation in the RSNNS library uses the mlp function for configuration and training. Configuration details are in Figure 23, Figure 24 and Figure 25.

**Figure 26:**
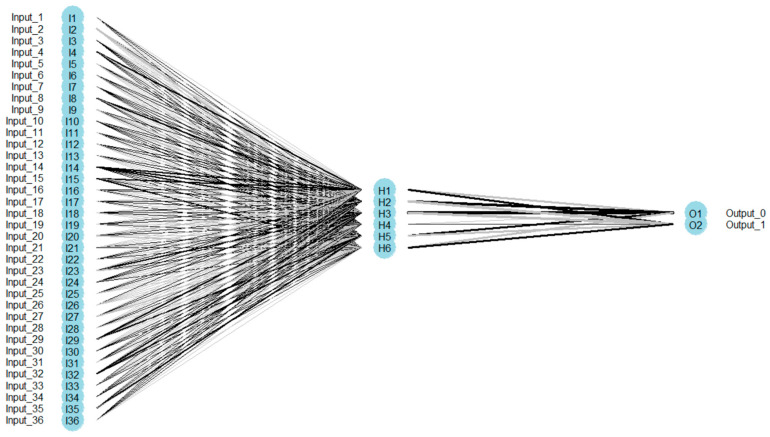
MLP network architecture—for vector of sums of subfields in the mask.

The implementation of the MLP network in the Keras library required a detailed list of layers in the code. Two layer_dense layers were used; the first one defines the hidden layer with the ReLU activation function, and the second one defines the output layer with the size 2 (two output categories) using the softmax activation function (Figure 26).

### 3.3. Convolution Neural Network

For an objective comparison of the Keras and MXNet libraries, the same convolution network architecture in both libraries was used at first, however in the MXNet library, training such a neural network was too slow. Thus, we designed our own architecture with a better learning time performance. The discussion about the results is provided in the next Section 4.

**Figure 27:**
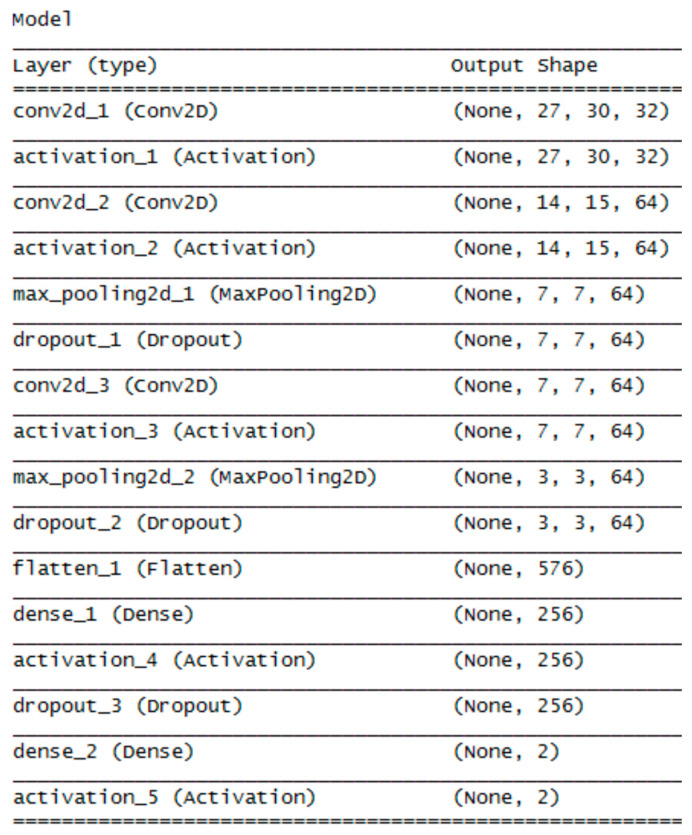
Architecture of the convolution neural network 1.

**Figure 28:**
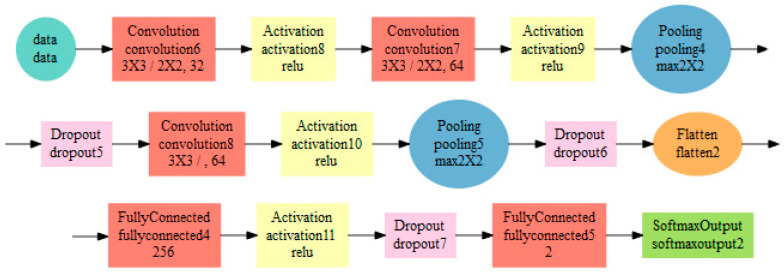
Architecture visualization of the convolution neural network 1.

The architecture of the convolution network 1 is shown in Figure 27 and visualized in Figure 28. The architecture includes a list of all layers and the size of output structures for both NN. Two pairs of convolution and pooling layers were used, the convolution being applied twice before the first pooling layer. The input image size was 56 × 60. The number of convolution filters was 32 at the beginning, in further convolution filters it rose to 64. A dropout was used between some layers to prevent overtraining of the neural network by deactivating a certain percentage of randomly selected neurons. At the end, the flatten layer was used to convert the resulting structure into a one-dimensional vector used as an input for a simple MLP network with one hidden layer containing 256 neurons.

Parameters of individual layers are shown in the diagram in Figure 28. For example, the convolution layer (red) contains a list of 3 × 3 - filter size, 3 × 3 - stride, 32 - number of filters.

**Figure 29:**
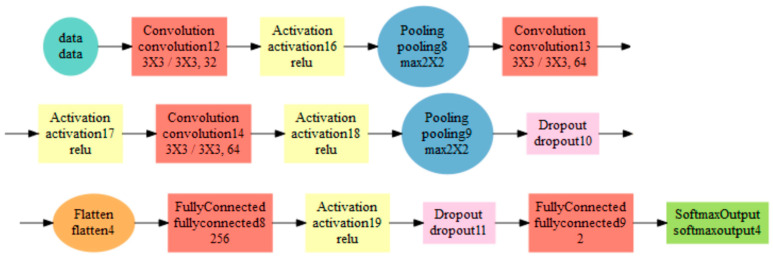
Architecture visualization of the Convolution neural network 2.

The architecture of the convolution network 2 is visualized in Figure 29. Two pairs of convolution and pooling layers were used, however in this case a double convolution occurs only in the second layer. There is also a difference in the design of the convolution, where the parameter stride (step of the filter) is 3,3. Dropout was used only in two places.

## 4. Results

This chapter presents results of code profiling, weld segmentation and evaluation of neural networks.

### 4.1. Code Profiling

**Figure 30:**
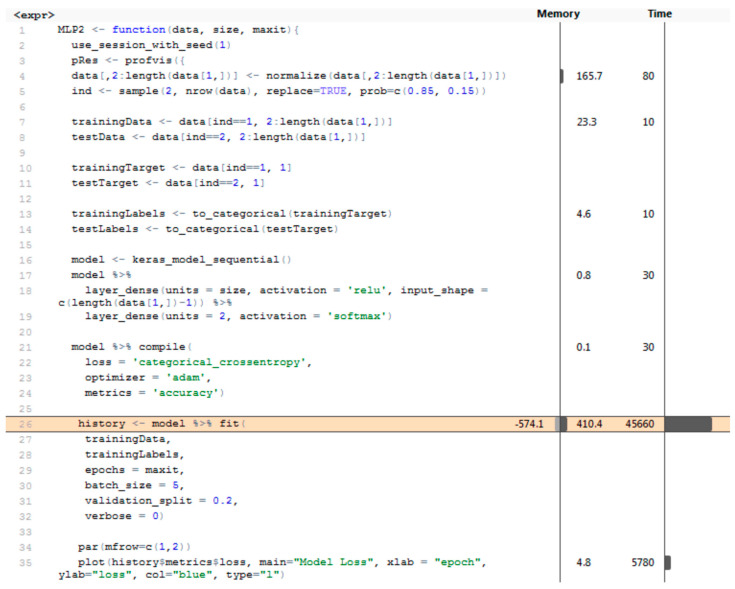
Example of profiling output using profvis.

Profiling was done using the profvis library at the level of the code line. The output is an interactive visualization using memory listing in MB and computing time in ms for each code line. The example can be seen in Figure 30.

Profiling was performed on a desktop computer with parameters listed in Table 1 (the graphic card was not used).

### 4.2. Results of Data Preparation and Segmentation

**Figure 31:**
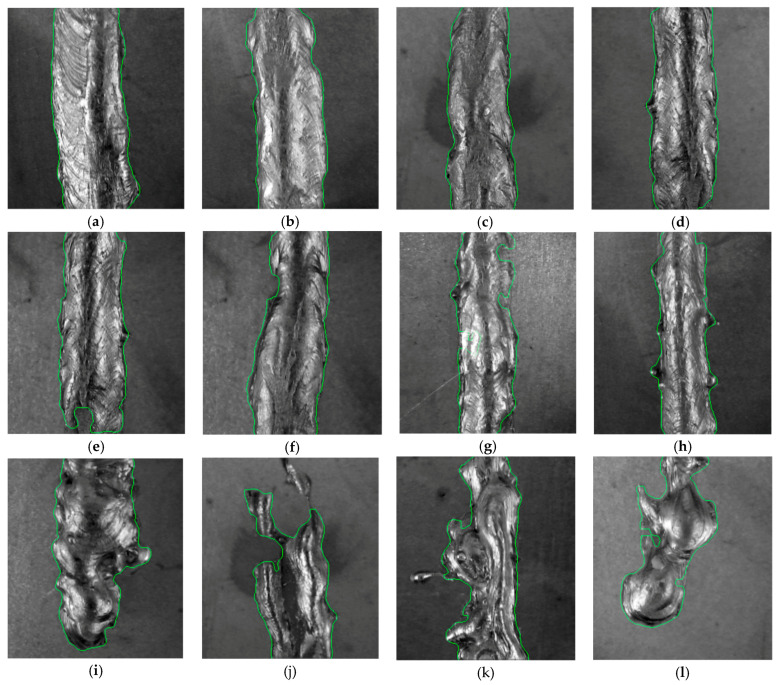

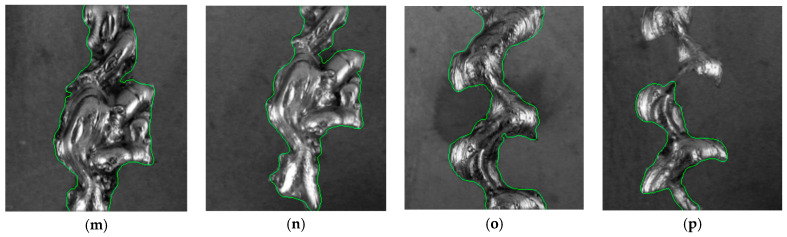
Examples of weld segmentation results (**a**–**p**).

Segmentation was successful for all tested weld samples. For some NOK defective welds which consisted of several parts or contained droplets, only the largest continuous weld surface was segmented, which was considered to be a correct segmentation for proposed methodology. Segmentation examples are shown in Figure 31.

**Figure 32:**
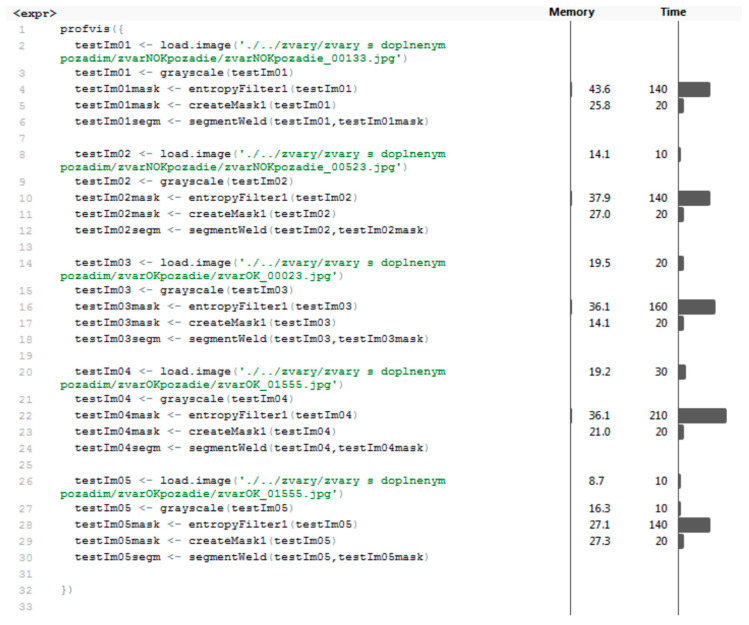
Results of segmentation process profiling.

The segmentation time is an important indicator in comparison of results. Results of profiling different parts of the segmentation process can be seen in Figure 32. Code profiling was carried out using a computer with the technical specification shown in Table 1.

**Figure 33:**
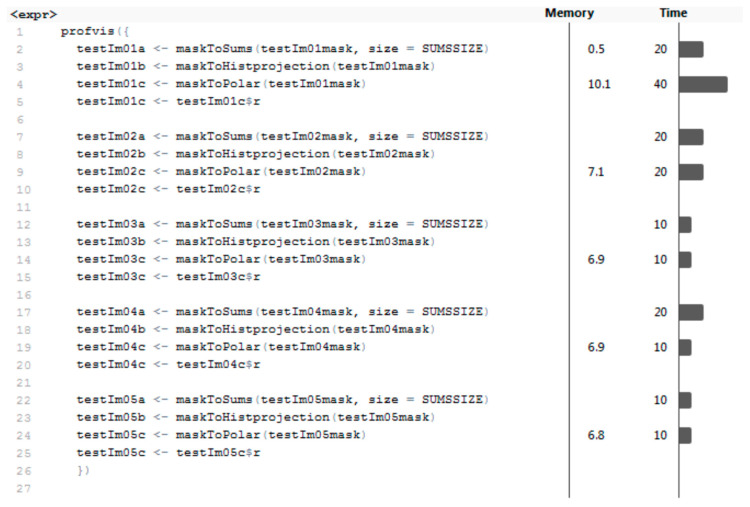
The profiling results of data transformation.

Segmentation was performed by concatenating the outputs from functions load.image, grayscale, entropyFilter, createMask, and segmentWeld. Almost all functions in this section of the program were performed very quickly (within 30 ms) except for the entropyFilter function, which took an average of 158 ms to be completed. This function is the most important part of the segmentation algorithm; the time was acceptable. The average time to complete the whole segmentation was 194 ms. The average amount of memory allocated was 74.76 MB. For MLP and RBF networks, the next step was to transform masks into feature vectors. The profiling results of functions performing three types of transformations can be seen in Figure 33.

The results show that these functions are optimal, taking up minimal memory and time. The mean values for computing the vector of sums of subfields in the mask are 16 ms and 0.1 MB; for the histogram projection vector, it is less than 10 ms and less than 0.1 MB (estimation of profiling tool, real values are immeasurably small). Values for the polar coordinates vector are 18 ms and 7.56 MB. Presented results are also shown in Table 2.

### 4.3. Criteria for Evaluation of Neural Network Results

**Figure 34:**
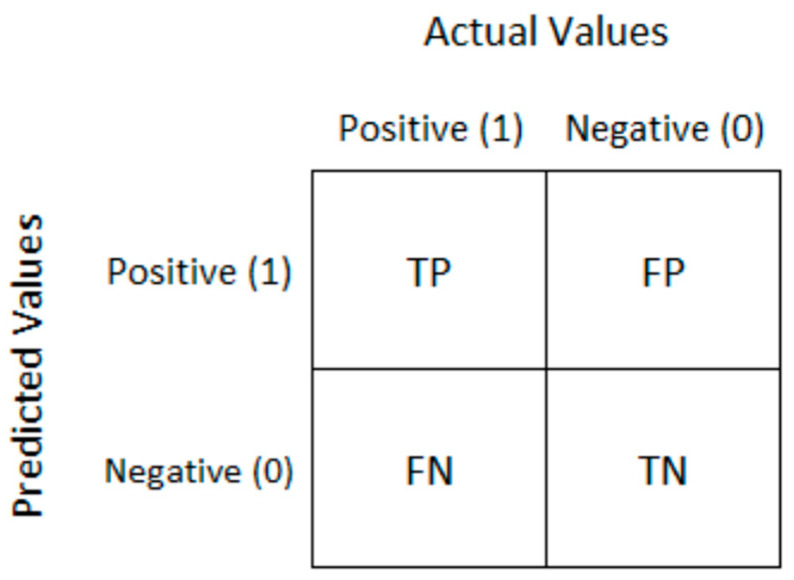
Confusion matrix.

As the main criterion for results evaluation the confusion matrix was chosen. The main diagonal of the confusion matrix contains the numbers of correctly classified samples, the antidiagonal contains the numbers of incorrectly classified samples; the smaller values in the antidiagonal, the more successful the prediction model. In a binary classification this matrix contains four values (Figure 34): TP—true positive; FP—false positive; FN—false negative; TN—true negative.

The accuracy was computed from the confusion matrix and is expressed as the ratio of correctly classified samples to all samples, see Equation (5) [20].

(5) $$Accuracy=\frac{\sum TP+\sum TN}{\sum all\;samples},$$

Accuracy is an objective criterion only if the FN and FP values are similar.

A more objective criterion for comparing results is the F-score. The *F-score* is calculated as the harmonic average of the precision and the recall (sensitivity) values [20], the best score corresponds to F-score = 1:

(6) $$Precision=\frac{\sum TP}{\sum TP+\sum FP},$$

(7) $$Recall=\frac{\sum TP}{\sum TP+\sum FN},$$

(8) $$F-score=\frac{2*Recall*Precision}{\sum TP+\sum FN\;Recall+Precision},$$

**Figure 35:**
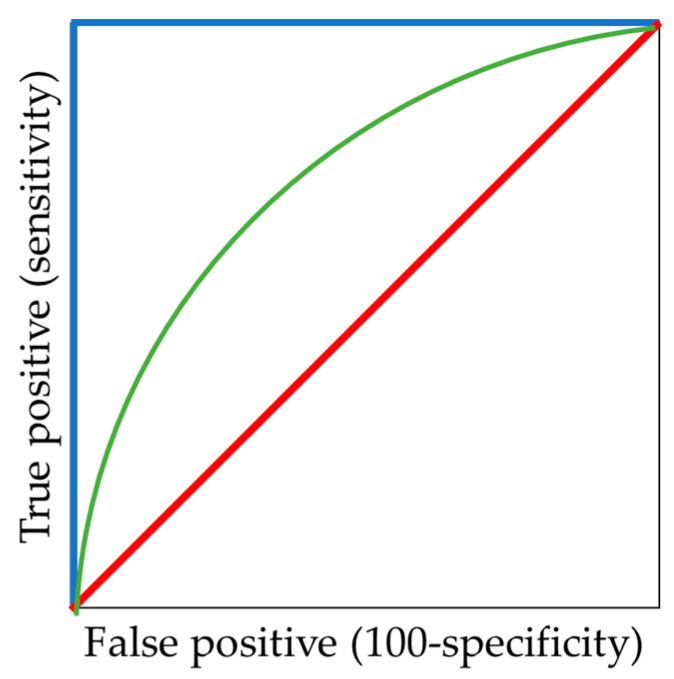
ROC curves: excellent (blue); good (green); worthless (red).

To visualize the success of neural network classification, the ROC (Receiver operating characteristics) curve was chosen. It shows the recall (sensitivity) value depending on the value 1-specificity at the variable threshold [20] (Figure 35):

(9) $$Specificity=\frac{\sum TN}{\sum TN+\sum FP},$$

The ROC curve for the best possible classifier is rectangular with the vertex [0,1].

### 4.4. Results of Neural Network Classificaton

We configured and tested neural networks for all data representations (in total 15 experiments). For a better clarity, the experiments results are labelled using labels from Table 3.

The first tests were carried out for RBF and MLP networks with input data formats according to Table 3. Resulting confusion matrices for RBF networks are as follows:

(10) $$\begin{array}{c} rbf-rsn-sum01=\left[\begin{array}{cc} 502 & 14\\ 15 & 433 \end{array}\right],\\ rbf-rsn-hpr02=\;\left[\begin{array}{cc} 434 & 0\\ 83 & 447 \end{array}\right],\\ rbf-rsn-pol03=\left[\begin{array}{cc} 435 & 0\\ 82 & 447 \end{array}\right], \end{array}$$

**Figure 36:**
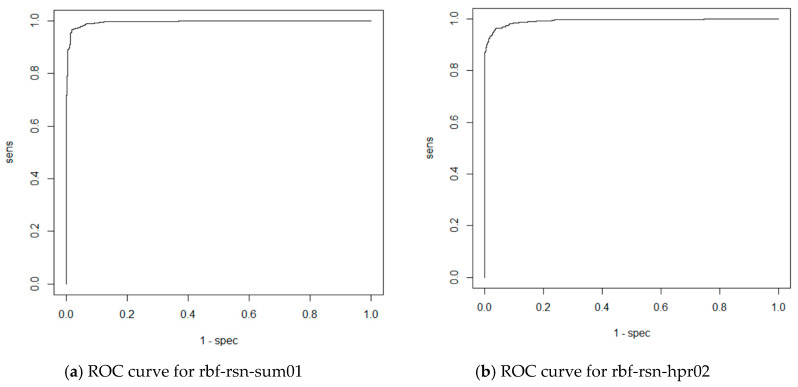

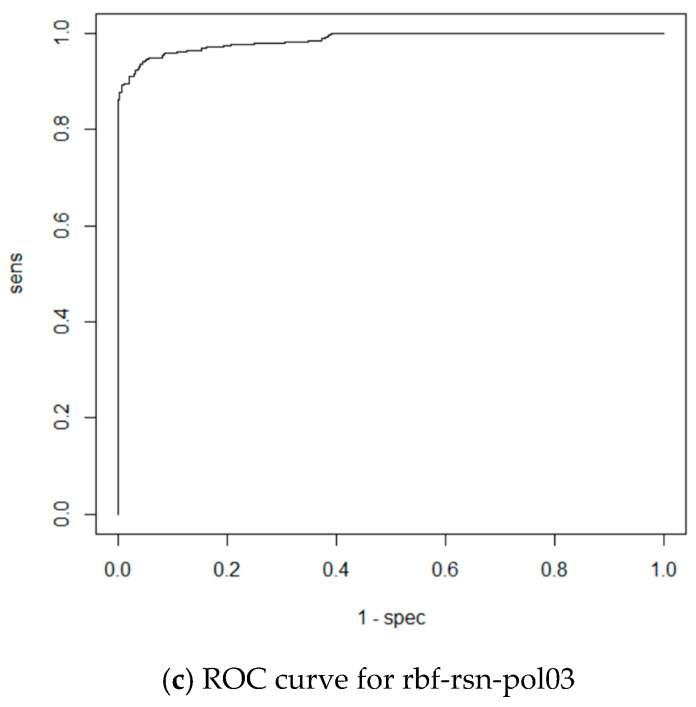
ROC curves for experiments with RBF networks.

From the matrices (10) it is evident that the RBF network performed bad when classifying NOK welds—they are often classified as OK. ROC curves of trained RBF networks are depicted in Figure 36.

**Figure 37:**
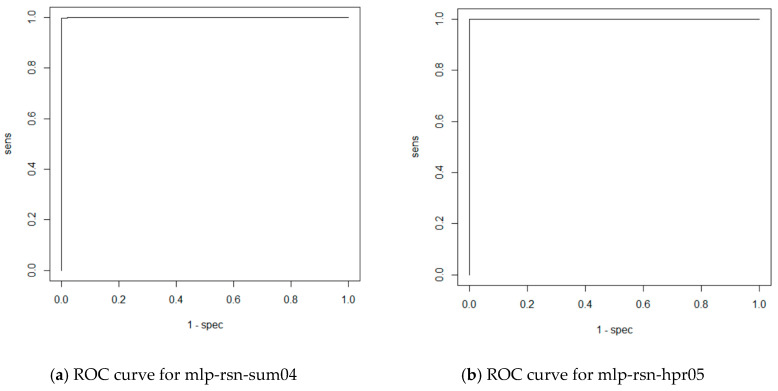

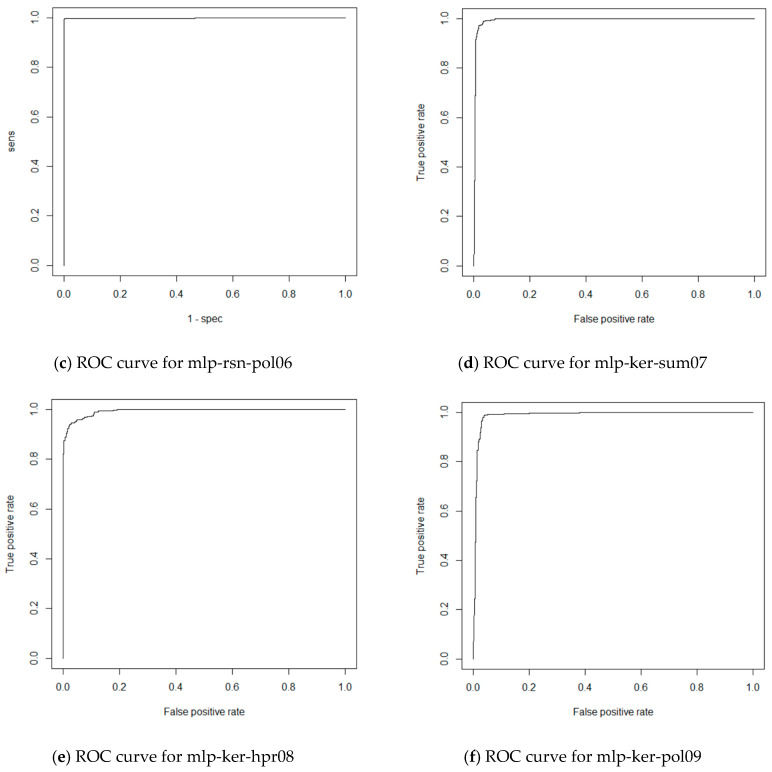
ROC curves for experiments with MLP networks.

ROC curves for MLP networks are depicted Figure 37 and Resulting confusion matrices are as follows:

(11) $$\begin{array}{c} mlp-rsn-sum04=\left[\begin{array}{cc} 516 & 1\\ 1 & 446 \end{array}\right],\\ mlp-rsn-hpr05=\left[\begin{array}{cc} 5017 & 0\\ 0 & 447 \end{array}\right],\\ mlp-rsn-pol06=\left[\begin{array}{cc} 514 & 1\\ 3 & 446 \end{array}\right], \end{array}$$

(12) $$\begin{array}{c} mlp-ker-sum07=\left[\begin{array}{cc} 517 & 15\\ 17 & 446 \end{array}\right],\\ mlp-ker-hpr08=\left[\begin{array}{cc} 511 & 2\\ 23 & 459 \end{array}\right],\\ mlp-ker-pol09=\left[\begin{array}{cc} 522 & 13\\ 12 & 448 \end{array}\right], \end{array}$$

The results show that the MLP implementation in the RSNNS library was more successful compared with the Keras library. The networks had no problem to classify correct (OK) or incorrect (NOK) welds. FP and FN values were approximately similar. The resulting calculated accuracy and F-scores shown in Table 4 describe the performance of the trained neural networks.

The results show that MLP networks are much more successful. Using default RBF initialization weights the RBF network less successful. From a practical point of view, MLP networks are more suitable for weld evaluation.

It was hard to compare the results for MLP networks, they provided similar results for all data representations. The RBF network achieved significantly better results in the vector of sums of subfields in the mask data representation.

It was found out, that using the same network configuration in the two libraries yields slightly different results. The implementation in the RSNNS library was almost 100% successful and therefore it was considered as the best candidate for practical use.

Training profiling for RSNN library was done next. Although training in the Keras library allocated less memory, the training time was several times longer than in case of the RSNNS library. Using vector of sums of subfields in the mask, the MLP network training time in RSNNS took less than one second, while using the Keras library was tens of seconds. The list of training profiling results is shown in Table 5.

Comparison of convolution neural nets was again based on the confusion matrices, ROC curves, accuracy and F-scores. The input of the networks were just images of welds without any filtration and masked welds without background (black background). Confusion matrices are as follows:

(13) $$\begin{array}{c} cnn-ker-ori10=\left[\begin{array}{cc} 534 & 1\\ 0 & 460 \end{array}\right],\\ cnn-mxn-ori12=\;\left[\begin{array}{cc} 559 & 8\\ 1 & 431 \end{array}\right],\\ cnn-mxn-ori14=\left[\begin{array}{cc} 498 & 0\\ 0 & 460 \end{array}\right], \end{array}$$

(14) $$\begin{array}{c} cnn-ker-seg11=\left[\begin{array}{cc} 534 & 0\\ 0 & 461 \end{array}\right],\;\\ cnn-mxn-seg13=\;\left[\begin{array}{cc} 558 & 0\\ 2 & 439 \end{array}\right],\\ cnn-mxn-seg15=\left[\begin{array}{cc} 498 & 0\\ 0 & 460 \end{array}\right], \end{array}$$

**Figure 38:**
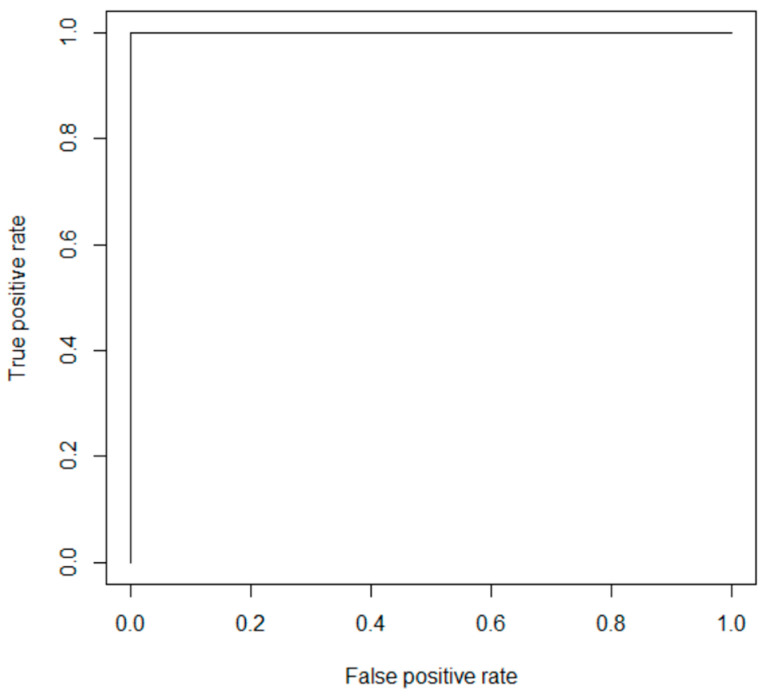
ROC curve for all convolution nets.

Classification error in convolution neural networks was minimal, therefore the ROC curve was evaluated as ideal for all experiments with indistinguishable differences. For all neural nets, the ROC curve was the same (Figure 38).

The resulting accuracy and F-scores along with the number of epochs needed to train the networks are listed in Table 6.

**Figure 39:**
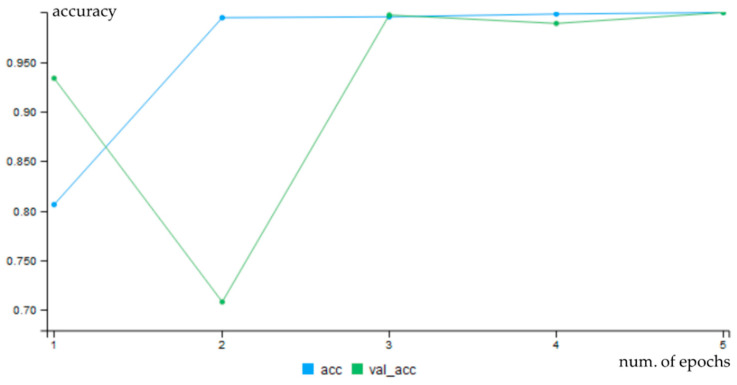
Progress of accuracy for cnn-ker-ori10.

**Figure 40:**
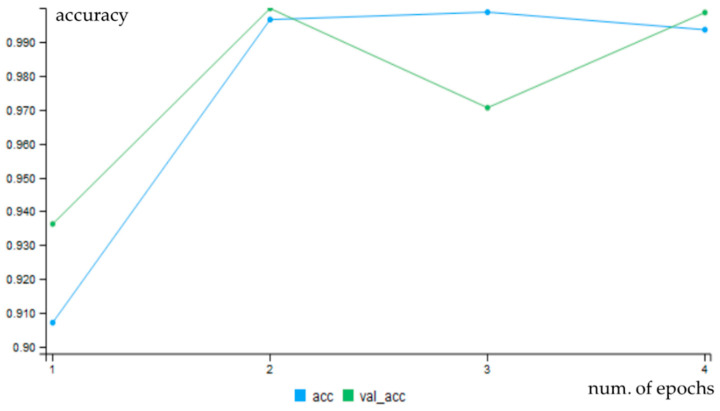
Progress of accuracy during epochs for cnn-ker-seg11.

For convolution networks, changes of accuracy after each epoch for both training (blue line) and validation data (green line) are shown in Figure 39. The charts show that training with non-segmented weld images started at a lower accuracy and the learning was slower (Figure 40).

**Figure 41:**
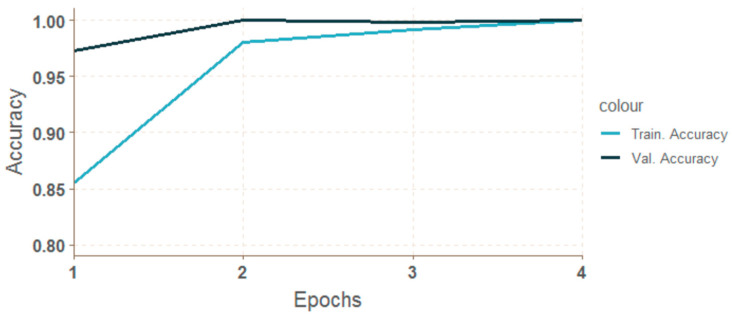
Progress of accuracy during epochs for cnn-mxn-ori14.

**Figure 42:**
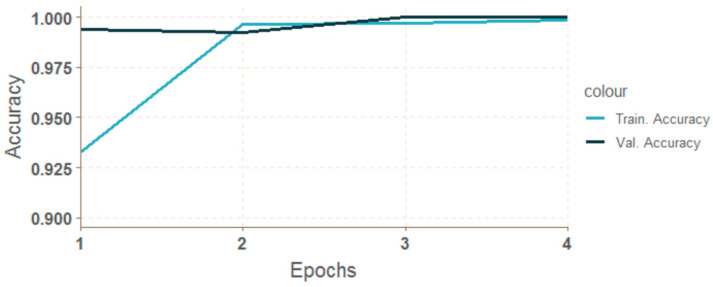
Progress of accuracy during epochs for cnn-mxn-seg15.

The progress of training for the Keras library was more uniform, without steps. The graphs can be seen in Figure 41 and Figure 42.

The success rate for all networks was higher than 99%. The decisive factor for comparison were the code profiling results shown in Table 7.

It can be concluded, that the network with the architecture shown in Figure 29 in Section 3.3 implemented using the MXNet library was the fastest. With a training time 12.170 ms and a 100% success also for non-segmented data it is considered the best choice for practical use.

Although the MLP network (mlp-rsn-sum04) was similarly successful and several times faster in training, the preparation of the representation in the form of the vector of sums of subfields in the mask took considerably more time. The number of training samples was approximately 5400, the average time to obtain a mask of one sample was 164 ms, and the vector calculation was 16 ms, in total 972 ms.

### 4.5. Profiling Single Weld Diagnostics

In practice, neural network training is not a frequent process. Usually, the network is trained once and then implemented for prediction. Therefore, at the end we decided to evaluate the prediction of one weld for the most successful models. The provided results represent the average of five independent tests. The list can be seen in Table 8 along with the average image preparation time and memory required to prepare the weld input image for the specific diagnostic model.

The diagnostic profiling results confirmed that the best solution was the classification of the weld using the convolution net with the architecture shown in Figure 29 in Section 3.3. The average image loading time and its 5× reduction took only 14 ms on average, and evaluation time was 14 ms.

## 5. Discussion

The aim of this paper was to develop a neural network based methodology to evaluate quality of welds. Several types of neural networks implemented in several software libraries were compared with respect to performance. It was necessary to prepare the data (images of welds) into a format suitable for neural network processing. For some types of networks (convolution) the input data preparation was minimal (segmentation or no segmentation), while for other networks (MLP, RBF), a sophisticated data preprocessing was required (filtering, equalizing and segmenting the image based on entropy). Each library required its own input data format which also had to be taken into account during programming. The main result of the paper is confirmation, that the convolutional neural networks can be used for weld quality evaluation without using image preprocessing and in case of using no segmentation, they can be used for evaluation not only weld metal but also adjected zones.

Neural networks were configured experimentally to achieve the best performance and the obtained results were compared. In all cases, neural networks implemented and trained using the proposed approach delivered excellent results with a success rate of nearly 100%. Thus, we can recommend any of the tested libraries to solve the weld quality evaluation problem. The best results were achieved using convolution neural networks which provided excellent results and with almost no pre-processing of image data required. The longer training time of these networks is acceptable in practical usage.

In summary, based on achieved experimental results, convolution neural networks have shown to be a promising approach for weld evaluation and will be applied in the future research dealing with evaluation of images in the real welding processes. The convolutional neural networks can be used for weld quality evaluation without using image preprocessing.

## Figures and Tables

**Table 1:** Technical specifications of PC.

**Operating System**	Windows 7 Professional 64-bit
**Processor**	Intel Core i7-2600 CPU @ 3,40 GHz
**Memory**	16 GB DDR3
**Disc**	Samsung SSD 850 EVO 500 GB

**Table 2:** Algorithms results for transform masks into feature vectors.

Data Interpretation	Time [ms]	Memory [MB]
the vector of sums of subfields in the mask	16	0.1
histogram projection vector	10	0.1
polar coordinates vector	18	7.56

**Table 3:** Labels of neural network experiment.

Test Label	Network Type	Library	Data Format
rbf-rsn-sum01	RBF	RSNNS	Subfields sum
rbf-rsn-hpr02	RBF	RSNNS	Histogram projection
rbf-rsn-pol03	RBF	RSNNS	Polar coordinates
mlp-rsn-sum04	MLP	RSNNS	Subfields sum
mlp-rsn-hpr05	MLP	RSNNS	Histogram projection
mlp-rsn-pol06	MLP	RSNNS	Polar coordinates
mlp-ker-sum07	MLP	Keras	Subfields sum
mlp-ker-hpr08	MLP	Keras	Histogram projection
mlp-ker-pol09	MLP	Keras	Polar coordinates
cnn-ker-ori10	CNN 1	Keras	Original
cnn-ker-seg11	CNN 1	Keras	Segmented
cnn-mxn-ori12	CNN 1	MXNet	Original
cnn-mxn-seg13	CNN 1	MXNet	Segmented
cnn-mxn-ori14	CNN 2	MXNet	Original
cnn-mxn-seg15	CNN 2	MXNet	Segmented

**Table 4:** Accuracy a F-score for RBF and MLP networks.

Test Label	Accuracy	F-Score
rbf-rsn-sum01	0.9699	0.9719
rbf-rsn-hpr02	0.9139	0.9127
rbf-rsn-pol03	0.9149	0.9139
mlp-rsn-sum04	0.9979	0.9981
mlp-rsn-hpr05	1.0000	1.0000
mlp-rsn-pol06	0.9959	0.9961
mlp-ker-sum07	0.9678	0.9700
mlp-ker-hpr08	0.9761	0.9761
mlp-ker-pol09	0.9766	0.9766

**Table 5:** Profiling of RBF and MLP networks training.

Test Label.	Time [ms]	Memory [MB]
rbf-rsn-sum01	6660	687.6
rbf-rsn-hpr02	42,530	775.6
rbf-rsn-pol03	32,080	752.3
mlp-rsn-sum04	850	769.8
mlp-rsn-hpr05	9890	653.7
mlp-rsn-pol06	17,270	672.0
mlp-ker-sum07	52,830	485.2
mlp-ker-hpr08	45,660	410.4
mlp-ker-pol09	46,420	401.9

**Table 6:** Accuracy and F-scores for convolution neural network experiments.

Test Label	Epochs	Accuracy	F-Score
cnn-ker-ori10	5	0.9990	0.9991
cnn-ker-seg11	4	1.0000	1.0000
cnn-mxn-ori12	6	0.9910	0.9920
cnn-mxn-seg13	3	0.9980	0.9982
cnn-mxn-ori14	4	1.0000	1.0000
cnn-mxn-seg15	4	1.0000	1.0000

**Table 7:** Code profiling results for designed convolution neural networks.

Test Label	Epochs	Time [ms]	Memory [MB]
cnn-ker-ori10	5	38,610	186.9
cnn-ker-seg11	4	30,660	180.0
cnn-mxn-ori12	6	119,630	4.7
cnn-mxn-seg13	3	82,580	2.6
cnn-mxn-ori14	4	12,170	157.9
cnn-mxn-seg15	4	11,850	3.7

**Table 8:** Profiling results for single weld diagnostics.

Test Label	Image Time Preparation [ms]	Diagnostic Time [ms]	Memory [MB]
mlp-rsn-sum04	210	20	0.2
mlp-rsn-hpr05	194	240	3.0
mlp-rsn-pol06	198	105	1.8
cnn-mxn-ori14	14	14	0.5
cnn-mxn-seg15	194	4	0.5

## References

[1] Akşit M. The Role of Computer Science and Software Technology in Organizing Universities for Industry 4.0 and beyond. Proceedings of the 2018 Federated Conference on Computer Science and Information Systems, FedCSIS 2018.

[2] Dahal S., Kim T., Ahn K. (2016). Indirect prediction of welding fume diffusion inside a room using computational fluid dynamics. Atmosphere.

[3] Huang W., Kovacevic R. (2011). A laser-based vision system for weld quality inspection. Sensors.

[4] Noruk J. (2001). Visual weld inspection enters the new millennium. Sens. Rev..

[5] Deng S., Jiang L., Jiao X., Xue L., Deng X. (2009). Image processing of weld seam based on beamlet transform. Hanjie Xuebao/Trans. China Weld. Inst..

[6] Deng S., Jiang L., Jiao X., Xue L., Cao Y. Weld seam edge extraction algorithm based on Beamlet Transform. Proceedings of the 1st International Congress on Image and Signal Processing, CISP 2008.

[7] Zhang X., Yin Z., Xiong Y. Edge detection of the low contrast welded joint image corrupted by noise. Proceedings of the 8th International Conference on Electronic Measurement and Instruments, ICEMI 2007.

[8] Hou X., Liu H. Welding image edge detection and identification research based on canny operator. Proceedings of the 2012 International Conference on Computer Science and Service Systems, CSSS 2012.

[9] Shen Z., Sun J. Welding seam defect detection for canisters based on computer vision. Proceedings of the 6th International Congress on Image and Signal Processing, CISP 2013.

[10] Liao Z., Sun J. Image segmentation in weld defect detection based on modified background subtraction. Proceedings of the 6th International Congress on Image and Signal Processing, CISP 2013.

[11] Khumaidi A., Yuniarno E.M., Purnomo M.H. Welding defect classification based on convolution neural network (CNN) and Gaussian Kernel. Proceedings of the 2017 International Seminar on Intelligent Technology and Its Application: Strengthening the Link between University Research and Industry to Support ASEAN Energy Sector, ISITIA 2017.

[12] Pandiyan V., Murugan P., Tjahjowidodo T., Caesarendra W., Manyar O.M., Then D.J.H. (2019). In-process virtual verification of weld seam removal in robotic abrasive belt grinding process using deep learning. Robot. Comput. Integr. Manuf..

[13] Chen J., Wang T., Gao X., Wei L. (2018). Real-time monitoring of high-power disk laser welding based on support vector machine. Comput. Ind..

[14] Wang T., Chen J., Gao X., Qin Y. (2017). Real-time monitoring for disk laser welding based on feature selection and SVM. Appl. Sci..

[15] Haffner O., Kucera E., Kozak S., Stark E. Proposal of system for automatic weld evaluation. Proceedings of the 21st International Conference on Process Control, PC 2017.

[16] Haffner O., Kučera E., Kozák Š. Weld segmentation for diagnostic and evaluation method. Proceedings of the 2016 Cybernetics and Informatics, K and I 2016—Proceedings of the the 28th International Conference.

[17] Haffner O., Kučera E., Kozák Š., Stark E. Application of Pattern Recognition for a Welding Process. Proceedings of the Communiation Papers of the 2017 Federated Conference on Computer Science and Information Systems, FedCSIS 2017.

[18] Haffner O., Kučera E., Bachurikova M. Proposal of weld inspection system with single-board computer and Android smartphone. Proceedings of the 2016 Cybernetics and Informatics, K and I 2016—Proceedings of the the 28th International Conference.

[19] Gajowniczek K., Ząbkowski T., Orłowski A. Comparison of decision trees with Rényi and Tsallis entropy applied for imbalanced churn dataset. Proceedings of the 2015 Federated Conference on Computer Science and Information Systems, FedCSIS 2015.

[20] Sokolova M., Lapalme G. (2009). A systematic analysis of performance measures for classification tasks. Inf. Process. Manag..

